# Abundance and Distribution of Microbial Cells and Viruses in an Alluvial Aquifer

**DOI:** 10.3389/fmicb.2017.01199

**Published:** 2017-07-11

**Authors:** Donald Pan, Jason Nolan, Kenneth H. Williams, Mark J. Robbins, Karrie A. Weber

**Affiliations:** ^1^School of Biological Sciences, University of Nebraska-Lincoln Lincoln, NE, United States; ^2^Department of Earth and Atmospheric Sciences, University of Nebraska-Lincoln Lincoln, NE, United States; ^3^Lawrence Berkeley National Laboratory Berkeley, CA, United States

**Keywords:** virus, bacteriophage, dissolved organic carbon, aquifer, subsurface, uranium, groundwater

## Abstract

Viruses are the most abundant biological entity on Earth and their interactions with microbial communities are recognized to influence microbial ecology and impact biogeochemical cycling in various ecosystems. While the factors that control the distribution of viruses in surface aquatic environments are well-characterized, the abundance and distribution of continental subsurface viruses with respect to microbial abundance and biogeochemical parameters have not yet been established. In order to begin to understand the factors governing virus distribution in subsurface environments, we assessed microbial cell and virus abundance in groundwater concurrent with groundwater chemistry in a uranium impacted alluvial aquifer adjoining the Colorado River near Rifle, CO. Virus abundance ranged from 8.0 × 10^4^ to 1.0 × 10^6^ mL^−1^ and exceeded cell abundance in all samples (cell abundance ranged from 5.8 × 10^4^ to 6.1 × 10^5^ mL^−1^). The virus to microbial cell ratio ranged from 1.1 to 8.1 and averaged 3.0 ± 1.6 with virus abundance most strongly correlated to cell abundance (Spearman's ρ = 0.73, *p* < 0.001). Both viruses and cells were positively correlated to dissolved organic carbon (DOC) with cells having a slightly stronger correlation (Spearman's ρ = 0.46, *p* < 0.05 and ρ = 0.54, *p* < 0.05; respectively). Groundwater uranium was also strongly correlated with DOC and virus and cell abundance (Spearman's ρ = 0.62, *p* < 0.05; ρ = 0.46, *p* < 0.05; and ρ = 0.50, *p* < 0.05; respectively). Together the data indicate that microbial cell and virus abundance are correlated to the geochemical conditions in the aquifer. As such local geochemical conditions likely control microbial host cell abundance which in turn controls viral abundance. Given the potential impacts of viral-mediated cell lysis such as liberation of labile organic matter from lysed cells and changes in microbial community structure, viral interactions with the microbiota should be considered in an effort to understand subsurface biogeochemical cycling and contaminant mobility.

## Introduction

Viruses have been identified in every environment where microorganisms are present, often equal to or exceeding microbial cell abundance (Suttle, 2005; Anderson et al., 2013; Knowles et al., 2016). Within the continental subsurface sediments (10^3^–10^9^ cm^−3^; Engelhardt et al., 2014; Pan et al., 2014; Yanagawa et al., 2014) and groundwater (10^5^–10^7^ mL^−1^; Kyle et al., 2008; Roudnew et al., 2012) viruses may outnumber cells *in situ* by as much as 225 to one in the subsurface (Engelhardt et al., 2014). Considering that the continental subsurface harbors an estimated one-third of all microbial life on Earth (Whitman et al., 1998; Kallmeyer et al., 2012), the impact of viruses in terrestrial subsurface biogeochemical cycling is of growing interest (Pan et al., 2014; Wilkins and Fredrickson, 2015). However, we know very little about the role viruses play in subsurface microbial ecology and biogeochemistry. Viruses are obligate intracellular parasites that use the host cell for replication and often lyse the host cell upon release into the environment. Lysogenic viruses may also exist as prophage integrated into host genomes but may be triggered back into a lytic life cycle. As a consequence, virus-mediated cell lysis has the potential to liberate DOC and other nutrients into the surrounding environment contributing to food webs and biogeochemical carbon cycling (Fuhrman, 1999; Wommack and Colwell, 2000; Middelboe and Lyck, 2002; Suttle, 2005, 2007; Weitz and Wilhelm, 2012).

While we recognize that viruses are abundant in aquifers and other subsurface environments, the factors which influence the distribution and abundance of viruses are poorly characterized. In surface aquatic environments, these factors are well characterized such as host cell abundance (Liang et al., 2014; Wigington et al., 2015) and productivity (Maranger and Bird, 1995; Clasen et al., 2008); however the abundance and distribution of continental viruses with respect to subsurface parameters such as microbial abundance and geochemical properties have not yet been established. Since virus replication depends on host cells, factors that alter host microbial growth and productivity will also have an impact on virus production. In surface aquatic microbial ecosystems, the abundance, distribution, and biogeochemical impact of viral infection are not homogenous, but often correspond to the distribution of nutrients accessible in the aqueous environment (Seymour et al., 2006; Dann et al., 2014, 2016; Wang et al., 2016). Chemical factors including DOC have been demonstrated to influence the activity of microorganisms (Peter et al., 2012) and are linked to the distribution of viruses in aquatic environments (Laybourn-Parry et al., 2001; Farnell-Jackson and Ward, 2003). Thus, the distribution of subsurface viruses may also be linked to factors that govern cell distribution, such as carbon, nutrients, and energy in the subsurface. Previous studies have shown that the addition of acetate and an electron acceptor, nitrate, to subsurface sediment stimulated the production of viruses (Pan et al., 2014), suggesting that carbon and electron acceptor availability can influence virus abundance. While stimulation of microbial activity increases virus production, little information exists regarding the distribution of viruses and organic carbon in the shallow subsurface.

Subsurface sediments are geochemically and physically heterogeneous due to deposition and burial of soil horizons and surface derived organic material. Deposition thus forms dispersed organic-rich lenses (Blazejewski et al., 2005, 2009) and is common within alluvial sedimentary environments (Blazejewski et al., 2009; Ricker et al., 2013; Chaopricha and Marín-Spiotta, 2014). As a unique facies type organic-rich deposits represent an important component of subsurface sedimentary systems and contribute to the generation of geochemically reduced zones or hotspots in the subsurface (McClain et al., 2003). These hotspots consist of high concentrations of sediment-associated organic matter in reduced zones that maintain microbial activity and contain elevated concentrations of highly reduced chemical species (Qafoku et al., 2009; Campbell et al., 2012). Together both surface derived and buried organic matter play a significant role influencing microbial activity and biogeochemistry, controlling metal/radionuclide mobility across the upper Colorado River basin (Baker et al., 2000; Janot et al., 2015). One such aquifer is the Rifle alluvial aquifer, a former U.S. Department of Energy uranium ore-processing site near the city of Rifle, CO. Storage of uranium mill tailings at the site resulted in a large resilient groundwater uranium plume (Zachara et al., 2013). Recent research indicates that organic carbon rich regions contribute to geochemically reduced zones that play a role in the persistence of the U plume retaining U as a reduced mineral phase (Campbell et al., 2012; Qafoku et al., 2014; Janot et al., 2015). Uranium reduction to an insoluble mineral form is largely mediated by microbial activity, and as such it is not surprising that the presence of viruses in groundwater within the uranium plume was revealed in metagenomic datasets obtained from this aquifer (Wrighton et al., 2012; Holmes et al., 2015). The activity of viruses has implications for microbially-mediated biogeochemical processes such as metal reduction by directly influencing active populations of metal-reducing microorganisms. While viruses have been identified at this site, studies have not elucidated the abundance and distribution of viruses in the aquifer with respect to host cell abundance and geochemistry. Here, we determined the spatial distribution of microbial cells and viruses in groundwater collected from the Rifle aquifer with respect to groundwater geochemical data. To our knowledge, this is the first report of the spatial distribution of total virus abundance in correlation with aquifer geochemistry. Due to the importance of biogeochemical cycling in subsurface systems and the subsequent impact on the fate and transport of contaminants, understanding factors that control host cell and virus distribution in subsurface systems can help elucidate subsurface biogeochemistry.

## Materials and methods

### Study area

**Figure 1:**
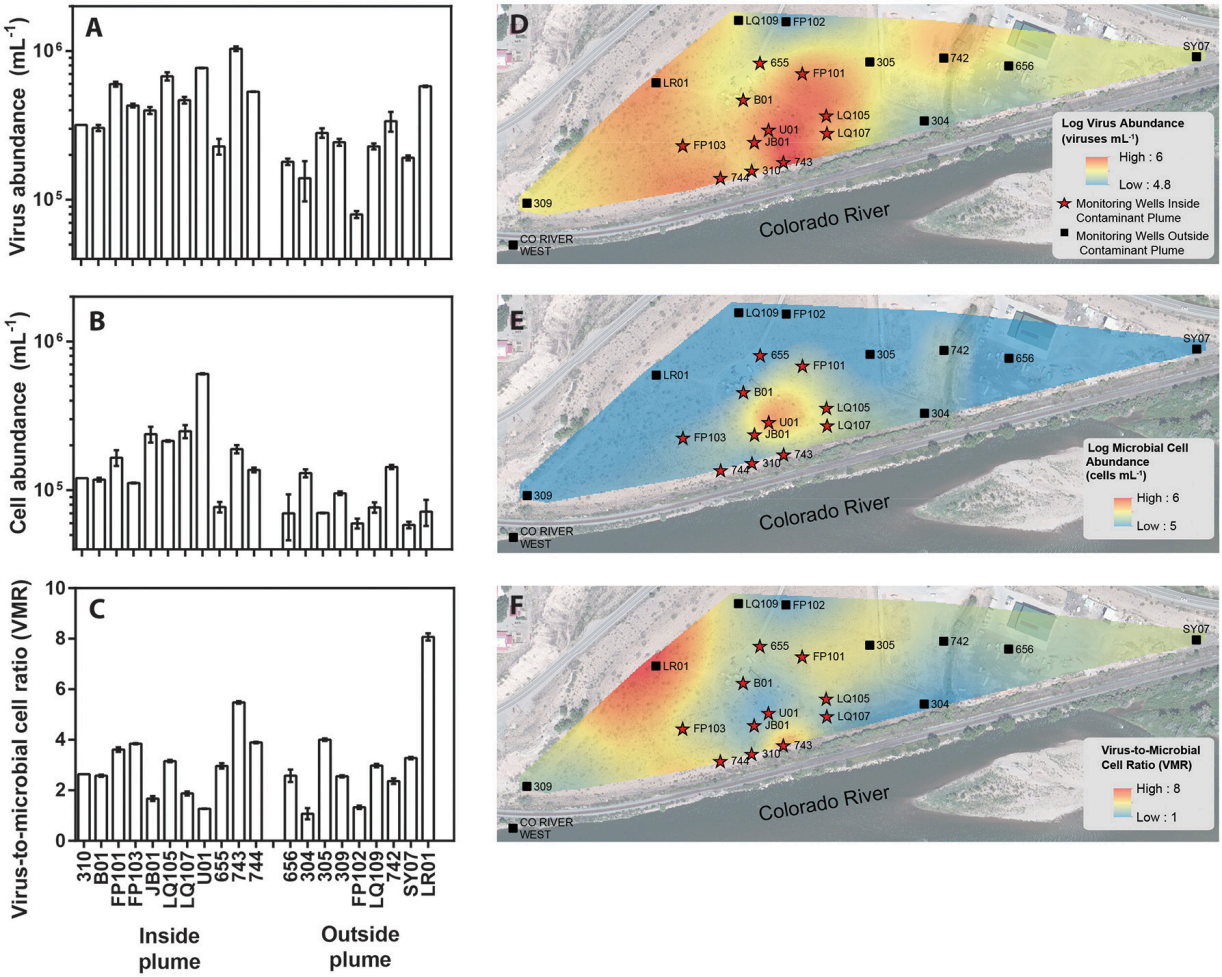
Location of monitoring wells within a uranium contaminated alluvial aquifer located 0.3 miles east of Rifle, Colorado (USA) and adjoining the Colorado River (Well 309 39.52842, −107.774426 and Well SY07 39.529335, −107.770864). Monitoring wells located inside the contaminant plume are denoted with red stars whereas monitoring wells located outside of the contaminant plume are denoted as black boxes. Groundwater virus abundance **(A)**, cell abundance **(B)**, and virus-to-microbial cell ratio (VMR) **(C)** data collected from monitoring wells across the alluvial aquifer. Error bars denoted standard error of measure for duplicate samples. Spatial interpolation of groundwater viruses **(D)**, cells **(E)**, and virus-to-microbial cell ratio **(F)** data collected from monitoring wells in the alluvial aquifer depicting spatial distribution. Color gradient from high (red) to low (blue) denotes interpolated values.

Groundwater was sampled using peristaltic pumps from 20 monitoring wells within a shallow (20–30 ft), unconfined alluvial aquifer adjoining the Colorado River located 0.3 miles east of Rifle, Colorado (USA) (Figure 1). Groundwater flows in a south-southwesterly direction and discharges into the Colorado River. The Holocene-age alluvial sediments consist of sandy gravel and gravelly sand containing silts and clays, characteristic of many alluvial aquifers. Distributed throughout the aquifer are also lenses of naturally reduced sediments containing reduced minerals and high concentrations of organic carbon originating from buried plant material (Campbell et al., 2012; Janot et al., 2015). Groundwater within the aquifer is typically suboxic (<1 mg L^−1^) and contains spatially varying concentrations of reduced chemical species such as Fe(II) (10–50 μM; Williams et al., 2011). Leaching of U from former stockpiles of ore and mill tailings stored at the site resulted in a persistent plume of groundwater with elevated U concentrations (>100 μg L^−1^; Zachara et al., 2013).

The major plume region has been described in prior studies conducted at the Rifle field site (Zachara et al., 2013). For the purpose of this study the boundaries of the plume region were defined where groundwater U concentrations exceeded 100 μg L^−1^. An elevated groundwater U concentration (171 μg L^−1^) was measured in well 656 in the eastern portion of the site, but groundwater in this region is geochemically distinct from the major plume with significantly lower groundwater DOC, DIC, and sulfate concentrations. Accordingly, well 656 is excluded from assignment to the major contaminant plume that is located at the center of the field site. Eleven monitoring wells within the uranium contaminated plume and nine monitoring wells outside the plume region were sampled in this study (Figure 1). Each well was approximately 6 m deep. Wells directly associated with regions impacted during prior experimental augmentation (Anderson et al., 2003) were excluded from this study. Further site details have been described elsewhere (Anderson et al., 2003; Vrionis et al., 2005; Zachara et al., 2013).

### Data collection and processing

Replicate groundwater samples (<50 mL) for geochemical analyses and enumeration of cells and viruses were collected from purged (12 L, ca. 1–1.5 well volumes) wells at a 5 m depth using a peristaltic pump. Groundwater was filtered through 0.45 μm PVDF filters to remove sediment particles for cell enumeration, and through 0.1 μm PVDF filters to remove cells for virus enumeration. In order to reduce background noise and improve filter clarity, samples for virus enumeration were treated with a nuclease, DNase I (10 U mL^−1^; Danovaro and Middelboe, 2010; Carreira et al., 2015). In this study, viruses are operationally defined as DNase resistant, SYBR Green I fluorescent particles smaller than 0.1 μm and larger than 20 nm. Using this operational definition, defective viruses, gene transfer agents, and other unknown particles may plausibly be included. A maximum cutoff size of 0.1 μm was selected to prevent inclusion of cells smaller than 0.2 μm as was identified by Luef and colleagues at the Rifle site (Luef et al., 2015). To avoid including viruses larger than 0.1 μm in the cellular fraction, a minimum cutoff size of 0.2 μm was used for cell enumeration. A comparison of the sub 0.2 μm fraction and the sub 0.1 μm fraction showed that there was no statistically signficant difference (Figure S1). Thus viruses and cells that are between 0.1 μm to 0.2 μm were not enumerated in this study. Aliquots (1 mL) were preserved for both virus and cell counts by adding glutaraldehyde to a final concentration of 0.5% and incubating 15–30 min at 4°C prior to freezing in liquid N_2_ (Brussaard, 2009). Samples were packed in dry ice and shipped to the University of Nebraska-Lincoln for storage at −80°C prior to enumeration.

Duplicate samples were thawed for enumeration by epifluorescence microscopy. Viruses were collected on Anodisc filters (0.02 μm), while cells were collected on black polycarbonate filters (0.2 μm). Between 0.5 and 1 mL of sample was passed through each filter. SYBR Green I (400x dilution from original stock) was used to stain the filters (15 min) for enumeration by epifluorescence microscopy. We note that because SYBR Green I binds to dsDNA more efficiently than ssDNA and RNA, the total virus count may be underestimated. After staining and drying, filters were mounted on slides with an anti-fading solution (50% glycerol, 50% phosphate buffered saline, 0.1% *p*-phenylenediamine). Background fluoresence in groundwater samples was minimal and did not interfere with enumeration (Figure S2). At least 10 fields or 200 particles were enumerated per filter (Patel et al., 2007). For each sample, duplicate field replicates were enumerated for cells and viruses. Blanks of TE buffer were routinely checked to confirm the lack of viral and microbial contaminants on filters.

Groundwater dissolved oxygen (DO) concentrations were measured *in situ* deploying multi-parameter sondes (YSI Inc., OH) into the well. Groundwater samples were directly filtered (0.45 μm PTFE) into glass vials for analysis of DOC/DIC while samples for anion analysis were filtered directly into HDPE vials. Vials were capped, leaving no headspace, and stored at 4°C prior to analyses (Williams et al., 2011). Aqueous anions (sulfate and nitrate) were measured by ion chromatography (ICS-2100 equipped with AS18 column, Dionex, CA; Kantor et al., 2013). DOC/DIC was measured by combustion catalytic oxidation and NDIR method using a Shimadzu Total Organic Carbon Analyzer (TOC-VCSH; Shimadzu, Corp.). Measurements of DOC will also include cell and viral biomass due to the cutoff used for defining the aqueous fraction (<0.45 μm), however cell and viral biomass do not make a significant portion of any DOC measurement in this study. Using upper limits of 149 fg of C per cell (Vrede et al., 2002) and 10^6^ cells mL^−1^, no more than 0.15 mg L^−1^ can come from cellular biomass, which constitutes only a minor fraction of the DOC measured in this study. Viruses, being orders of magnitude smaller than cells, are a negligible component of total measured DOC.

### Data analyses

Spatial interpolation (ArcGIS, Desktop Release 10.1, Environmental Systems Research Institute, Redlands, CA) was used to geographically represent the distribution of viruses and cells as well as geochemical parameters (DOC, DIC, sulfate, dissolved Fe, dissolved Mn, nitrate, and pH) across the alluvial aquifer (Nolan and Weber, 2015). Spline interpolation method (from the ArcGIS Spatial Analyst extension) was selected from among three interpolation methods (kriging, inverse distance weighting, spline) as it resulted in the lowest residual error (Akkala et al., 2010).

Correlation analyses and statistical comparisons were conducted in GraphPad Prism 5.0.3 (GraphPad Software). Significance level was defined at *p* < 0.05. Analyses involving cell and virus abundances were conducted on log-transformed values. All correlations between all measured parameters were calculated by the Spearman rank correlation method. Statistical comparisons between parameters within the major plume region and outside were conducted by *t*-test.

## Results and discussion

### Groundwater geochemistry and virus and microbial cell distribution and abundance

**Figure 2:**
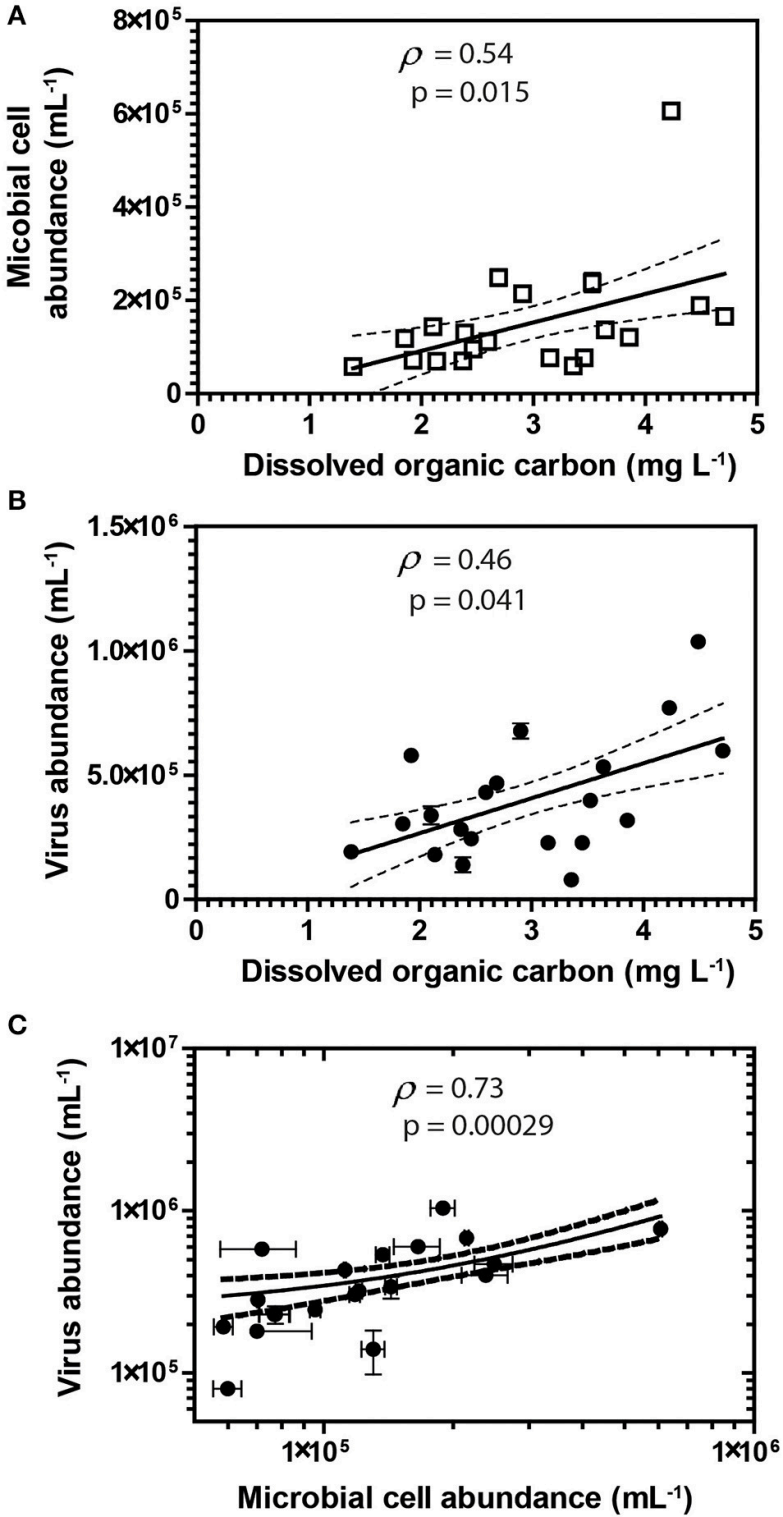
Spearman's rank correlations between DOC and abundances of cells **(A)** and viruses **(B)**. Spearman's rank correlation between viruses and cells **(C)** in groundwater samples. Error bars represent the standard deviation of the mean of duplicate measurements. Error bars not visible are smaller than the symbol. Dashed lines represent the 95% confidence interval for the lines of regression presented in the figure.

Groundwater virus abundance ranged from 8.0 × 10^4^ to 1.0 × 10^6^ viruses mL^−1^ (Figures 1A,D) and exceeded cell abundance (range 6.0 × 10^4^ to 6.1 × 10^5^ cells ml^−1^; Figures 1B,E) in all 20 monitoring wells. These total abundance values of viruses enumerated in groundwater were similar to results obtained from other groundwater sites (Kyle et al., 2008; Roudnew et al., 2012). Virus abundance in this shallow aquifer was strongly correlated to cell abundance (ρ = 0.73, *p* < 0.001) (Figure 2C) and is consistent with prior studies comparing virus and cell abundance in aquatic and sedimentary environments including lakes (Maranger and Bird, 1995; Bettarel et al., 2006; de Araújo and Godinho, 2009; Barros et al., 2010), marine waters (Alonso et al., 2001; Pereira et al., 2009), marine surface sediments (Danovaro and Serresi, 2000), marine subsurface sediment (Bird et al., 2001; Engelhardt et al., 2014), and deep granitic groundwater (Kyle et al., 2008). The virus-to-microbial cell ratio (VMR) in this shallow aquifer ranged from 1.1 to 8.1 and averaged 3.0 ± 1.6 (mean ± S.D., *n* = 20; Figures 1C,F). VMR range observed in groundwater collected from the Rifle aquifer is consistent with another shallow aquifer ranging from 0.4 to 6.1 (Roudnew et al., 2012) but is slightly less than has been observed in deep aquifers (average VMR of 12; Kyle et al., 2008). However, the VMR was notably lower than was measured in water collected from the Colorado River (VMR = 33; Table S1). It should be noted that the VMR in the Colorado River reported in this study is consistent with results from other river systems (Mathias et al., 1995; Jiao et al., 2006; Luef et al., 2007). This result is not surprising as total virus abundance in the river water sample exceeds virus abundance in groundwater. The lower VMR in groundwater relative to the river water sample could be a result of adsorption of viruses to the aquifer alluvium. Free (planktonic) viruses in the groundwater were enumerated in this study so any viruses produced that were adsorbed onto the aquifer alluvium (including clays and reactive minerals) would have been excluded resulting in a lower total virus abundance in the groundwater. The adsorption of viruses to minerals such as clays and iron oxides (Hewson and Fuhrman, 2003; You et al., 2005; Kernegger et al., 2009; Nieto-Juarez and Kohn, 2013) is recognized to reduce planktonic virus abundance. Previous studies have found a correlation between viral abundance and microbial activity in marine waters (Corinaldesi et al., 2003). In subsurface sediments, stimulation of microbial activity was found to result in an increase in VMR (Pan et al., 2014). Thus, the differences in viral abundance/VMR may indicate differences in microbial activity between groundwater and surface water. Together these are plausible reasons that may explain the difference in the abundance of viruses between the river water and groundwater samples.

**Figure 3:**
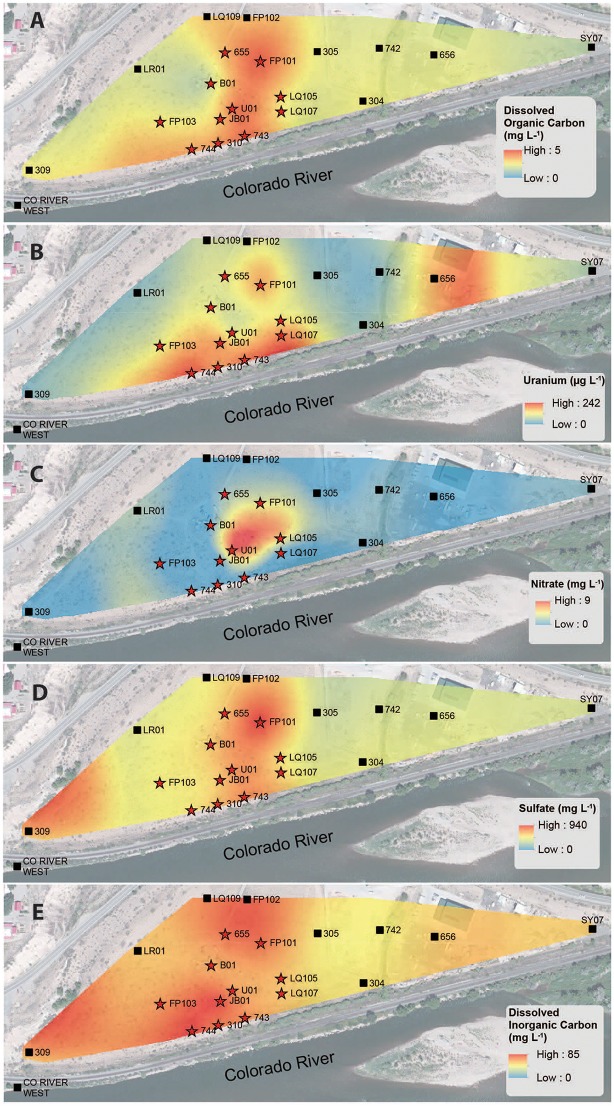
Spatial interpolation of groundwater DOC **(A)**, uranium **(B)**, nitrate **(C)**, sulfate **(D)**, and DIC **(E)** data collected from monitoring wells in the alluvial aquifer depicting spatial distribution. Color gradient from high (red) to low (blue) denotes interpolated concentrations.

A correlation between microbial cell abundance, virus abundance, and groundwater geochemistry was observed within the aquifer. Microbial cell abundance in groundwater was significantly higher (Unpaired *t*-test, *p* < 0.01) within the uranium plume (2.0 × 10^5^ ± 1.4 × 10^5^ cells mL^−1^, mean ± S.D., *n* = 11) compared to cell abundance in groundwater outside of the plume (8.6 × 10^4^ ± 3.1 × 10^4^ cells mL^−1^, mean ± S.D., *n* = 9; Table 1, Figures 1B,E). Cell abundance had a strong positive correlation to groundwater DOC (Spearman's ρ = 0.54, *p* < 0.05), U (Spearman's ρ = 0.51, *p* < 0.05), and sulfate concentrations (Spearman's ρ = 0.47, *p* < 0.05). However, it should be noted that groundwater DOC concentrations were also higher within the uranium plume (3.45 ± 0.88 mg L^−1^; mean ± S.D., *n* = 11), relative to the concentrations measured outside (2.36 ± 0.60 mg L^−1^; mean ± S.D., *n* = 9) of the uranium plume (Table 1, Figure 3A). Elevated concentrations of DOC may arise from buried organic carbon identified at the site which have been demonstrated to be responsible for persistence of the U plume (Campbell et al., 2012; Janot et al., 2015; Boye et al., 2017). Groundwater DOC and U concentrations were also correlated with virus abundance (Spearman's ρ = 0.46, *p* < 0.05 and ρ = 0.46, *p* < 0.05, respectively; Figures 2A,B, Table 2). Similar to the distribution of cells in the aquifer, virus abundance in the uranium plume region was statistically higher (Unpaired *t*-test, *p* < 0.01), 5.2 × 10^5^ ± 2.4 × 10^5^ viruses mL^−1^ (mean ± S.D., *n* = 11), relative to the abundance of viruses outside of the region, 2.5 × 10^5^ ± 1.4 × 10^5^ viruses mL^−1^ (mean ± S.D., *n* = 9; Table 1, Figures 1A,D). While virus and cell abundance in groundwater were positively correlated to groundwater DOC concentration, VMR did not exhibit a statistically significant correlation to any geochemical factor (Table 2, Figures 2A,B).

**Table 1:** Comparison of biotic and geochemical parameters in samples collected from monitoring wells located inside and outside of the contaminant plume.

	**Inside plume (mean ± S.D.) (*n* = 11)**	**Outside plume (mean ± S.D.) (*n* = 9)**	***t*-test *p*-value**
Uranium (μg L^−1^)	157.5 ± 47.90	63.15 ± 45.62	0.0003^***^
Dissolved Organic Carbon (DOC) (mg L^−1^)	3.45 ± 0.88	2.36 ± 0.60	0.0055^**^
Dissolved Inorganic Carbon (DIC) (mg L^−1^)	69.8 ± 9.9	56.9 ± 12.5	0.0194^*^
Sulfate (mg L^−1^)	630.2 ± 134.5	451.5 ± 125.8	0.0071^**^
Electrical conductivity (μS/cm)	2,468 ± 366	1961 ± 444	0.0118^*^
Cell abundance (mL^−1^) (log)	5.24 ± 0.24	4.91 ± 0.14	0.0021^**^
Virus abundance (mL^−1^) (log)	5.68 ± 0.19	5.34 ± 0.24	0.0026^**^
Virus-to-microbial cell ratio (VMR)	3.00 ± 1.20	3.14 ± 2.06	0.8544
pH	7.37 ± 0.10	7.47 ± 0.14	0.0676
DO (mg L^−1^)	0.83 ± 1.38	0.49 ± 0.99	0.5438
Iron (mg L^−1^)	2.22 ± 2.96	1.03 ± 1.57	0.2899
Manganese (mg L^−1^)	0.78 ± 0.47	0.40 ± 0.34	0.0644
Nitrate (mg L^−1^)	1.76 ± 2.93	0.49 ± 0.67	0.2188

Stars indicate levels of significance (

* *P < 0.05*,

** *P < 0.01*,

*** *P < 0.001)*.

**Table 2:** Spearman's rank correlation analysis between measured biotic and geochemical parameters.

	**Virus abundance**	**Cell abundance**	**Virus-to-microbial cell ratio (VMR)**	**Dissolved Organic Carbon (DOC)**	**Dissolved Inorganic Carbon (DIC)**	**Electrical conductivity (EC)**	**pH**	**Dissolved Oxygen (DO)**	**Nitrate**	**Sulfate**	**Uranium**	**Iron**	**Manganese**
Virus abundance	–												
Cell abundance	0.726^***^	–											
VMR	0.382	−0.250	–										
DOC	0.460^*^	0.537^*^	−0.008	–									
DIC	0.179	0.202	0.012	0.777^***^	–								
EC	0.435	0.573^**^	−0.202	0.657^**^	0.400	–							
pH	−0.133	−0.131	0.109	−0.264^**^	−0.559^**^	−0.099	–						
DO	0.008	−0.055	−0.031	−0.280	−0.131	−0.248	−0.307	–					
Nitrate	0.325	0.213	0.012	0.032	−0.184	0.186	0.009	0.264	–				
Sulfate	0.384	0.466^*^	−0.159	0.771^***^	0.688^***^	0.830^***^	−0.381	0.298	0.047	–			
Uranium	0.459^*^	0.505^*^	0.126	0.620^**^	0.547^*^	0.462^*^	−0.153	−0.138	−0.262	0.439	–		
Iron	−0.011	0.344	−0.208	0.277	0.344	0.050	0.044	−0.369	−0.628^**^	0.231	0.374	–	
Manganese	0.045	0.239	0.036	0.514^*^	0.573^**^	0.332	0.011	−0.505^*^	−0.504^*^	0.468^*^	0.579^**^	0.726^***^	–

Stars indicate the level of significance (

* *P < 0.05*,

** *P < 0.01*,

*** *P < 0.001)*.

While there is the potential for hyporheic intrusion of DOC from the Colorado River, elevated DOC concentrations were not observed in samples collected from wells located near the river, nor were other proxies for river water incursion into the aquifer, such as low electrical conductivity (data not shown). While we did not test lability of the DOC, correlations with cell (Spearman's ρ = 0.46, *p* < 0.05) and virus abundance (Spearman's ρ = 0.54, *p* < 0.05) strongly suggest that DOC was sufficiently bioavailable to stimulate microbial activity. Microbial activity in groundwater is often stimulated by inputs of DOC (Baker et al., 2000; Sobczak and Findlay, 2002; Findlay et al., 2003; Foulquier et al., 2011; Li et al., 2012). The presence of bioavailable DOC and available electron acceptors may thus provide sufficient energy for stimulation of microbial respiration. Because viruses are reliant on metabolically active hosts for replication, microbial host energy availability favors the production of viruses. Host cell metabolic activity and growth rate has been directly demonstrated to increase virus adsorption rate and decrease the period of time between viral infection and lysis of the host for lytic viruses (Hadas et al., 1997). Lysogenic bacteriophage have also been demonstrated to respond to host cell metabolic activity; control of the lytic and lysogenic pathway is controlled by levels of cAMP, with high energy conditions favoring lysis (Hong et al., 1971; Rolfe et al., 1973). In addition, chronic infections, in which viruses are released without lysis of the host, also produce greater numbers of viruses under higher energy conditions (Brown and Dowell, 1968). This was recently demonstrated in a series of alluvial aquifer sediment microcosms where additions of an energy source, acetate, and electron acceptor, nitrate, not only resulted in the oxidation of organic carbon, but also significant virus production (Pan et al., 2014). This result is consistent with prior studies conducted in surface aquatic environments in which correlation between virus abundance and DOC has been observed (Laybourn-Parry et al., 2001, 2013; Auguet et al., 2005; Holmfeldt et al., 2010; Säwström and Pollard, 2012). Inputs of organic carbon increase microbial activity (Peter et al., 2012), and organic-rich regions are also inferred to have elevated microbial activities (Campbell et al., 2012). As a result elevated microbial activity would result in enhanced virus production and hence higher virus abundance within the plume. Viral production and expression of virus-related genes following acetate biostimulation has been demonstrated previously at the Rifle site (Holmes et al., 2015). Given that stimulated microbial activity will result in the production of viruses (Pan et al., 2014), the elevated abundance of viruses in the plume correlated with organic carbon suggests that there is ongoing microbial activity and virus production in the aquifer.

The consumption of organic carbon in this aquifer has been linked to the reduction of molecular oxygen, nitrate, iron, manganese, uranium, and sulfate as well as fermentation reactions (Wrighton et al., 2012; Anantharaman et al., 2016). While suboxic conditions (DO < 1 mg L^−1^) predominated throughout most of the plume, oxic conditions were identified in two wells within the center of the plume region: LQ105 (4.3 mg L^−1^) and U01 (2.6 mg L^−1^) (Table S2). Dissolved Fe and Mn concentrations suggested the presence of reduced Fe (Fe(II)) and Mn (Mn(II)) and were also substantially lower within these two wells (Table S2, Figure S3). Thus lower Fe and Mn concentrations may be due to oxidative precipitation of Fe or Mn oxide minerals or lack of metal reduction due to oxic conditions in the groundwater. The highest dissolved Fe and Mn concentrations were found in wells located along the central portion of the site closest to the Colorado River (744, 310, 743, JB01, LQ107, 304) (Figure S3). Groundwater U concentrations ranged from 26.5 μg L^−1^ to 7.4 mg L^−1^, largely localized to the center of the site (Figure 3B). Nitrate was also low to undetectable in most of the wells throughout the floodplain with the exception of the two oxic monitoring wells LQ105 and U01 (5.89 mg L^−1^ and 8.25 mg L^−1^, respectively) (Table S2, Figure 3C). This may reflect operative nitrification processes or lack of denitrification due to oxic conditions in these wells. These geochemical conditions indicate a substantially different redox environment, potentially due to intrusion of dissolved oxygen or nitrate at the capillary fringe (Williams and Oostrom, 2000). The oxic monitoring wells U01 and LQ105 also contained some of the highest cell and virus abundances in the major plume region (Figure 1). This may be expected because O_2_ mediated respiration is expected to support greater cell abundance and hence greater virus abundance. Sulfate concentrations averaged 630.2 ± 134.5 mg L^−1^ (mean ± S.D., *n* = 11) within the plume and 451.5 ± 125.8 mg L^−1^ outside of the contaminant plume (mean ± S.D., *n* = 9) (Unpaired *t*-test *P* < 0.05; Table 1, Figure 3D). In addition, DIC was also higher within the plume (69.8 ± 9.9 mg L^−1^, mean ± S.D., *n* = 11) compared to outside (56.9 ± 12.5 mg L^−1^, mean ± S.D., *n* = 9) (Table 1, Figure 3E). Sulfate could also serve as a potential electron acceptor and has been implicated in the generation and precipitation of reduced sulfur phases such as framboidal pyrite, mackinwite, and greigite (Qafoku et al., 2009; Janot et al., 2015) within the aquifer. Elevated virus abundance in the plume region is consistent with prior results demonstrating that bacterial sulfate reduction rates were correlated with viral abundance and distribution in estuarine sediments (Middelboe et al., 2003). As such microbial metabolisms could thus be supported by the dynamic changes in redox conditions that are associated with the influx of oxidants into a carbon-rich reduced system.

### Implications of viral activity on microbially-mediated subsurface biogeochemical cycling

Here we demonstrate that viruses, cells, and DOC are enriched in the major plume region of the Rifle aquifer. The widespread distribution of viruses at the Rifle aquifer is consistent with prior suggestions that viruses may play a potential role in influencing biogeochemical cycling at the site (Wrighton et al., 2014; Holmes et al., 2015). Interactions between organic carbon, cells, and viruses may be important in riparian aquifers across the upper Colorado River basin where buried organic matter plays an important role in mediating biogeochemical cycles and metal/radionuclide sequestration. Organic carbon availability can promote microbial activity (Baker et al., 2000; Sobczak and Findlay, 2002; Findlay et al., 2003; Foulquier et al., 2011; Li et al., 2012) and, in turn, virus production, which is reflected by elevated virus abundances. Thus, organic rich sediments may potentially represent regions of increased viral activity as a response to higher microbial metabolic activity.

Within the Rifle aquifer, virus mediated cell lysis has been suggested to contribute biologically available organic carbon or to suppress certain taxa responsible for biogeochemically important reactions at the site (Wrighton et al., 2014; Holmes et al., 2015). In addition to lysis, lysogeny is another possible life cycle. Recent proposed models of lysogeny have suggested that at high and low host cell densities lysogenic life cycles may be favored, however with the cell densities encountered in this study (10^5^–10^6^ cells/mL), lytic kill-the-winner dynamics are suggested to be favored (Knowles et al., 2016). Lysis of active members of the microbial community and liberation of organic carbon can both influence biogeochemical cycling. Together these impacts have biogeochemical implications for the long-persisting groundwater U plume located in the Rifle aquifer and other similar aquifers within the upper Colorado River basin. The increase in bioavailable carbon would accelerate cell turnover rates and organic carbon liberation (Middelboe et al., 1996; Noble and Fuhrman, 1999; Middelboe and Lyck, 2002; Eissler et al., 2003), creating labile particulate and dissolved organic carbon thereby providing a source of biologically available carbon (Xu et al., 2013, 2014) subsequently controlling the community from the bottom up. The liberated carbon can increase respiration rates of heterotrophic bacteria (Middelboe and Lyck, 2002), which may include U-reducing bacteria responsible for the precipitation and immobilization of U(IV) (Anderson et al., 2003; Chang et al., 2005; Williams et al., 2011). Alternatively, viruses may also depress rates of biogeochemical transformations by infecting and lysing the organisms responsible for important organic carbon dependent subsurface processes such as denitrification (Burt et al., 1999) or metal reduction (Holmes et al., 2015). Further research is necessary to explore the role that viruses play mediating microbial processes underpinning reactions responsible for the fate and transport of metals/radionuclides impacting groundwater quality.

## Author contributions

DP and KAW contributed to laboratory and statistical analyses of data. KHW and MR contributed to field sample collection and geochemical analyses. JN contributed to the spatial interpolation of data. DP and KAW contributed to the experimental design and data interpretation. All authors contributed to writing of the manuscript.

### Conflict of interest statement

The authors declare that the research was conducted in the absence of any commercial or financial relationships that could be construed as a potential conflict of interest.
