# Supplementary material for: Abundance and Distribution of Microbial Cells and Viruses in an Alluvial Aquifer
Source: Front Microbiol. 2017 Jul 11;8:1199. doi: 10.3389/fmicb.2017.01199 (PMC5504356; doi:10.3389/fmicb.2017.01199)
Supplement: Supplementary file 1 [file DataSheet1.docx]

Supplementary Material

**Abundance and Distribution of Microbial Cells and Viruses in an Alluvial Aquifer**

Donald Pan ^1†^, Jason Nolan ^2^, Kenneth H. Williams ^3^, Mark J. Robbins ^3^, Karrie A. Weber ^1,2^*

^1^School of Biological Sciences, University of Nebraska-Lincoln, Lincoln, NE, USA

^2^Department of Earth and Atmospheric Sciences, University of Nebraska-Lincoln, Lincoln, NE, USA

^3^Lawrence Berkeley National Laboratory, Berkeley, CA, USA

^†^Present address: Department of Subsurface Geobiological Analysis and Research, Japan Agency for Marine-Earth Science and Technology, 2-15 Natsushima-cho, Yokosuka, Kanagawa, 237-0061, Japan

*** Correspondence:** Karrie A. Weber, School of Biological Sciences, University of Nebraska-Lincoln, 348 Manter Hall, 1104 T St., Lincoln, NE, 68588-0118, USA.

kweber@unl.edu

**Supplementary Figures**


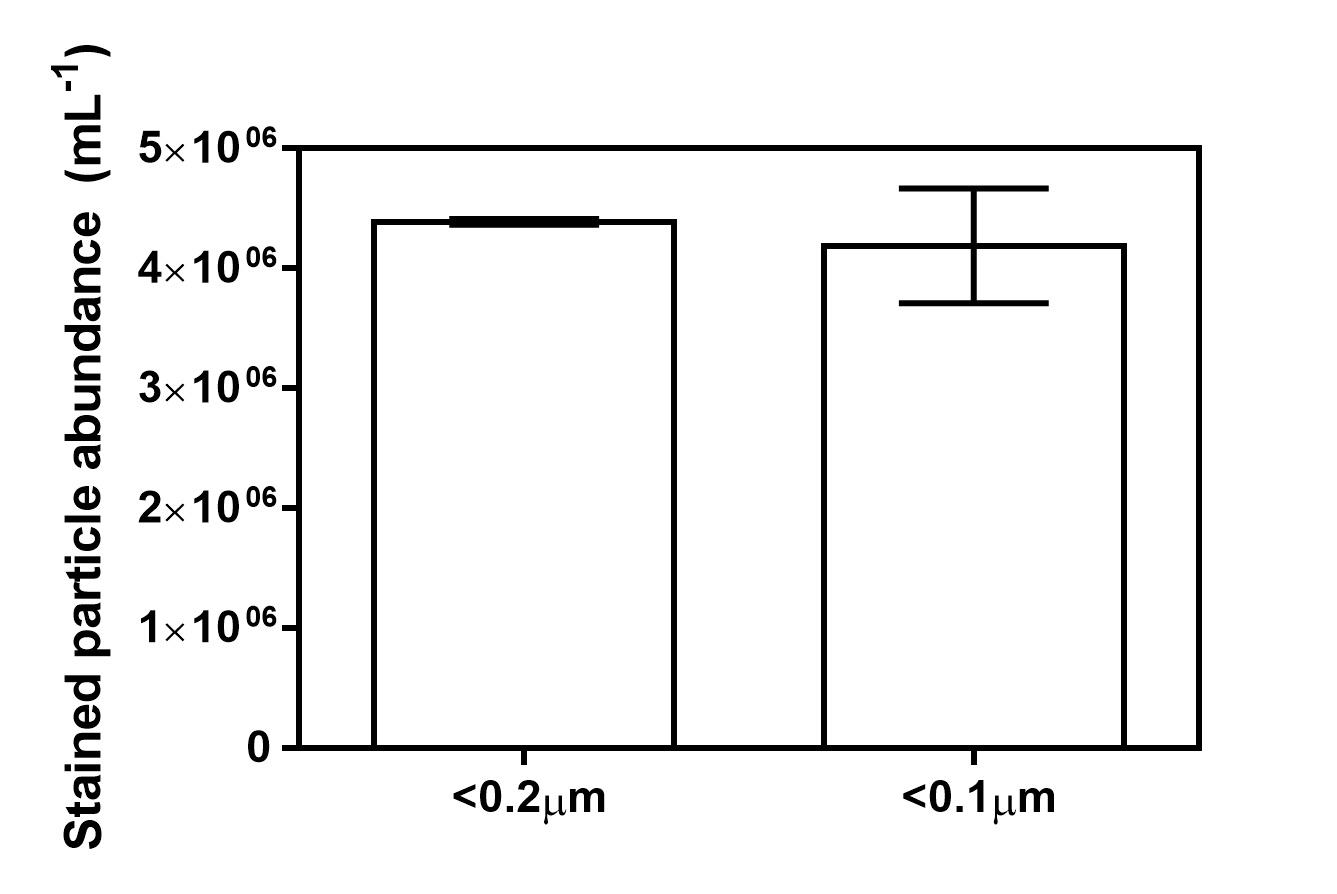


**Supplementary Figure S1**. Enumeration of groundwater collected from the Rifle aquifer filtered through 0.2 µm (PVDF) and 0.1 µm filters (PVDF) via epifluorescence microscopy revealed similar particle abundances after SYBR Green I staining (unpaired *t*-test *p*-value=0.7189). In order to avoid enumeration of cells smaller than 0.2 µm in the viral fraction, we functionally defined viruses as 0.1 µm or smaller. Error bars represent the standard of error of duplicate samples.

**Supplementary Figure S2**.
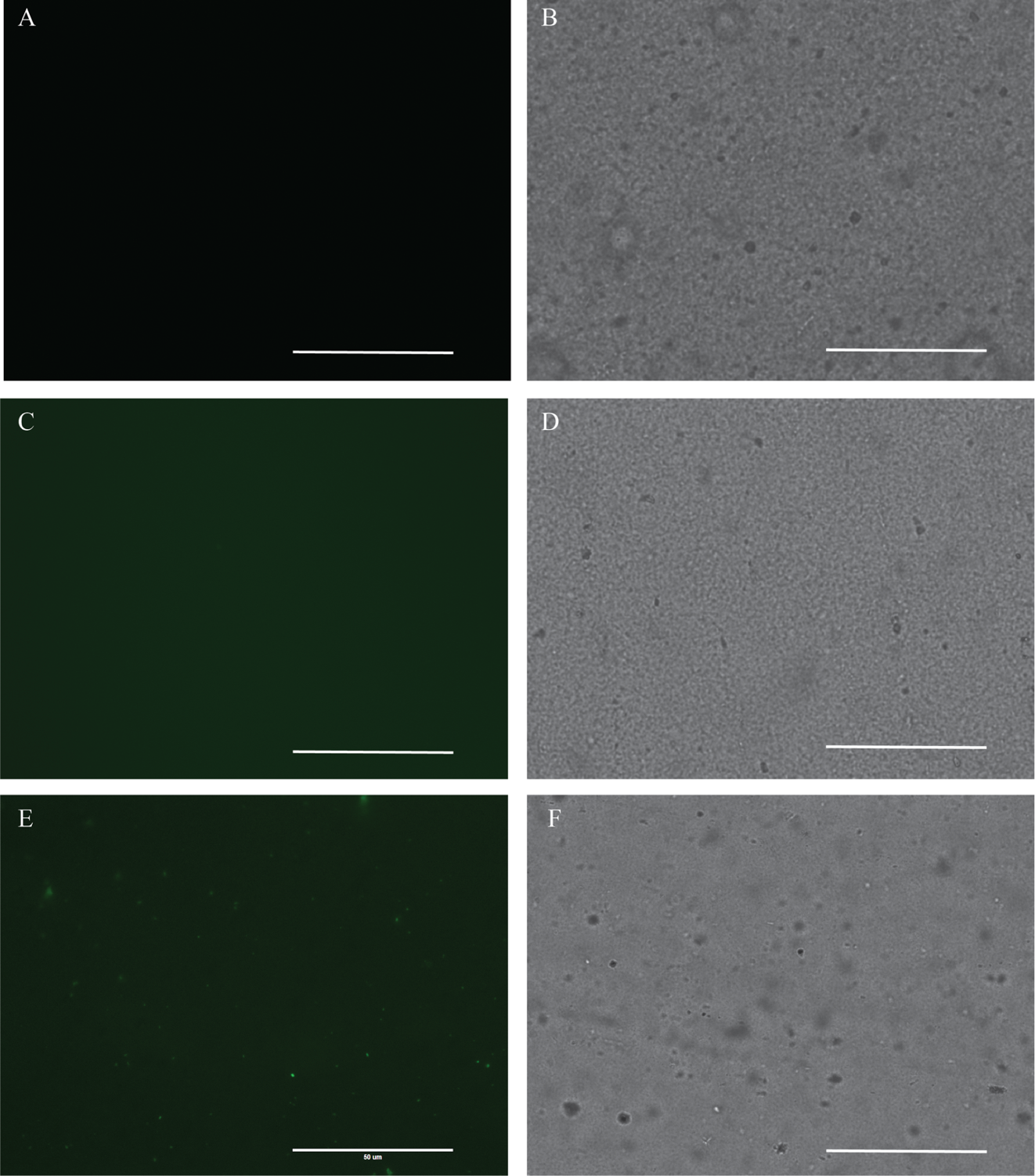
Groundwater samples collected from monitoring wells with dissolved uranium concentrations above 200 µg/L (well 743) were analyzed via epifluorescence microscopy in an effort to determine the impact of groundwater geochemistry on background fluorescence. Groundwater samples collected from monitoring well 743 were filtered through a 0.02 µm filter, then applied to a 0.02 µm Anodisc filter, and stained with SYBR Green I (**A**. and **B**.) and compared to similar preparations with TE Buffer (**C.** and **D.**). Another sample from the same well was filtered through a 0.1 µm filter and applied to a 0.02 µm Anodisc filter and stained with SYBR Green I (**E**. and **F**.). Epifluorescence micrographs of groundwater filtered through a 0.02 µm filter (**A**.) and 0.1 µm filter (**E**.) did not yield a high background relative to the background observed with 0.02 µm filtered TE buffer (**C**.). Particulate matter was visible in addition to stained viruses in the 0.1 µm filtered fraction (**E.**). Visible light micrographs of the filter (**B**., **D**., and **F**.) correspond to the epifluorescence micrographs on the left (**A**., **C**., and **E**.). Scale bar is equal to 50 µm.


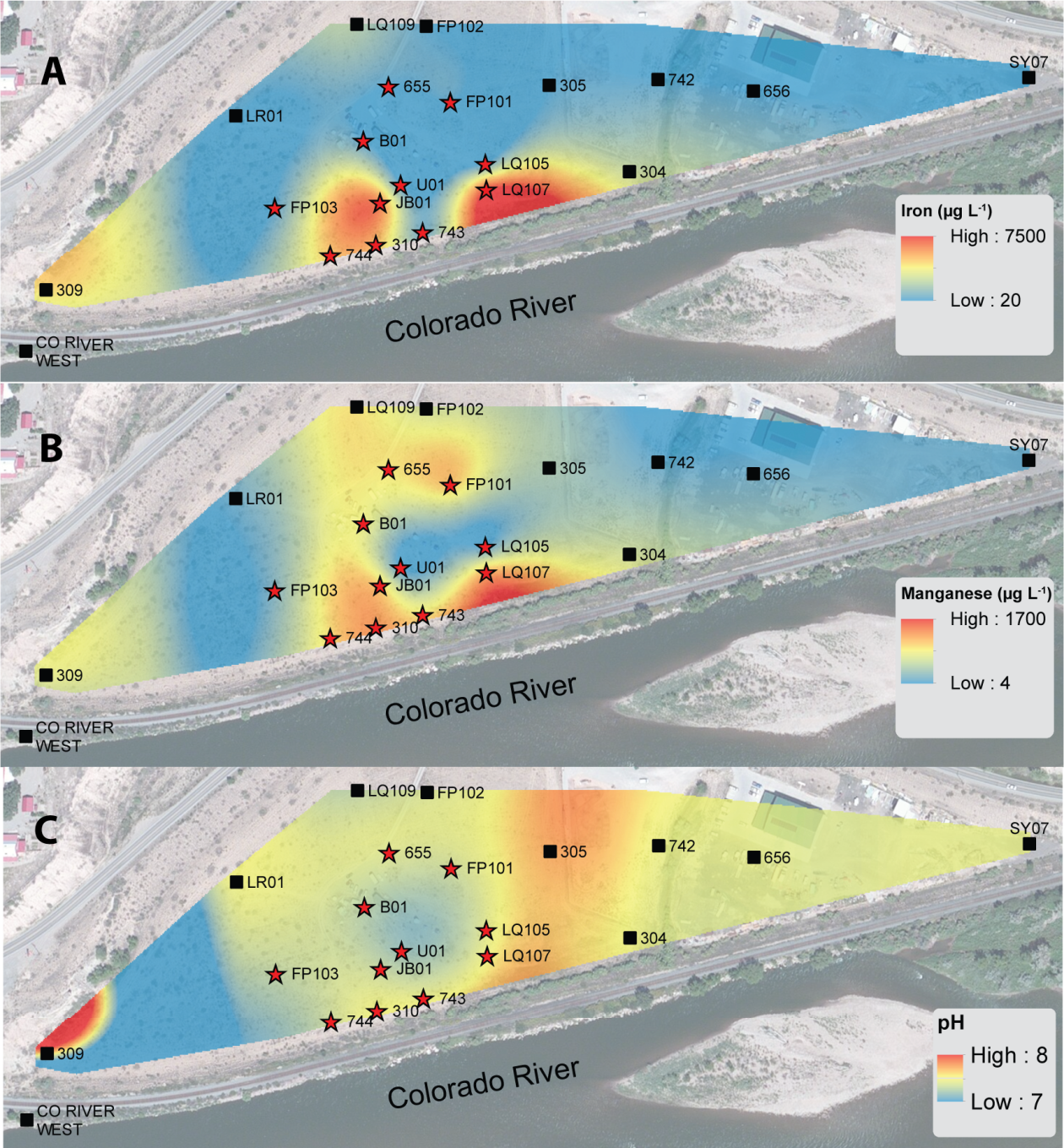


**Supplementary Figure S3**. Interpolation of iron **(A.)**, manganese **(B.)**, and pH **(C.)** data collected from monitoring wells in the alluvial aquifer depicting spatial distribution. Color gradient from high (red) to low (blue) denotes interpolated values. Monitoring wells located inside the contaminant plume are denoted with red stars whereas monitoring wells located outside of the contaminant plume are denoted as black boxes.

**Supplementary Tables**

**Supplementary Table S1.** Virus abundance, cell abundance, and virus-to-cell ratio from sampled wells.

| **Well** | **Viral abundance (mL^-1^)** | **Cell abundance (mL^-1^)** | **Virus-to-cell ratio (VCR)** |
| --- | --- | --- | --- |
| Within plume | | | |
| 310 | 3.19x10^5^ ± 8.78x10^3^ | 1.21x10^5^ ± 2.11x10^3^ | 2.64 ± 0.03 |
| B01 | 3.04x10^5^ ± 1.46x10^4^ | 1.18x10^5^ ± 3.37x10^3^ | 2.58 ± 0.06 |
| FP101 | 5.99x10^5^ ± 2.26x10^4^ | 1.66x10^5^ ± 2.02x10^4^ | 3.61 ± 0.13 |
| FP103 | 4.30x10^5^ ± 1.32x10^4^ | 1.12x10^5^ ± 6.32x10^2^ | 3.84 ± 0.03 |
| JB01 | 3.99x10^5^ ± 2.11x10^4^ | 2.38x10^5^ ± 2.91x10^4^ | 1.67 ± 0.13 |
| LQ105 | 6.78x10^5^ ± 4.28x10^4^ | 2.15x10^5^ ± 2.53x10^3^ | 3.16 ± 0.06 |
| LQ107 | 4.68x10^5^ ± 2.30x10^4^ | 2.49x10^5^ ± 2.53x10^4^ | 1.88 ± 0.11 |
| U01 | 7.70x10^5^ ± 3.95x10^3^ | 6.06x10^5^ ± 5.07x10^3^ | 1.27 ± 0.01 |
| 655 | 2.29x10^5^ ± 2.78x10^4^ | 7.71x10^4^ ± 6.26x10^3^ | 2.96 ± 0.15 |
| 743 | 1.04x10^6^ ± 3.80x10^4^ | 1.89x10^5^ ± 1.22x10^4^ | 5.48 ± 0.07 |
| 744 | 5.34x10^5^ ± 1.88x10^3^ | 1.37x10^5^ ± 5.06x10^3^ | 3.89 ± 0.04 |
| Outside plume | | | |
| 656 | 1.80x10^5^ ± 8.78x10^3^ | 6.99x10^4^ ± 2.38x10^4^ | 2.58 ± 0.34 |
| 304 | 1.40x10^5^ ± 4.23x10^4^ | 1.30x10^5^ ± 8.01x10^3^ | 1.07 ± 0.31 |
| 305 | 2.81x10^5^ ± 2.05x10^4^ | 7.03x10^4^ ± 3.88x10^2^ | 4.00 ± 0.07 |
| 309 | 2.44x10^5^ ± 1.32x10^4^ | 9.55x10^4^ ± 2.74x10^3^ | 2.55 ± 0.06 |
| FP102 | 7.97x10^4^ ± 4.39x10^3^ | 5.98x10^4^ ± 4.55x10^3^ | 1.33 ± 0.09 |
| LQ109 | 2.29x10^5^ ± 1.02x10^4^ | 7.68x10^4^ ± 6.16x10^3^ | 2.98 ± 0.09 |
| 742 | 3.38x10^5^ ± 5.12x10^4^ | 1.43x10^5^ ± 4.64x10^3^ | 2.36 ± 0.15 |
| SY07 | 1.91x10^5^ ± 7.32x10^3^ | 5.84x10^4^ ± 2.95x10^3^ | 3.28 ± 0.06 |
| LR01 | 5.80x10^5^ ± 6.95x10^3^ | 7.18x10^4^ ± 1.43x10^4^ | 8.07 ± 0.20 |
| Surface Water | | | |
| Colorado River | 4.09x10^6^ ± 6.06x10^4^ | 1.24x10^5^ ± 8.43x10^3^ | 33.01 ± 0.07 |

**Supplementary Table S2.** Concentrations of various groundwater parameters from sampled wells. The upper and lower limit of the range of measured values is represented by green and red respectively.

| **Well** | **Dissolved Oxygen**  **(mg L^-1^)** | **Fe(II) (µg L^-1^)** | **Electrical Conductivity (µS/cm)** | **Sulfate (mg L^-1^)** | **Nitrate (mg L^-1^)** |
| --- | --- | --- | --- | --- | --- |
| 304 | 0.063 | 2562.57 | 2039 | 418.8 | 0.68 |
| 305 | 0.384 | 52.66 | 2060 | 351.6 | 0.00 |
| 309 | 0.025 | 4365.38 | 2431 | 706.0 | 0.00 |
| 310 | 0.047 | 5067.71 | 2383 | 618.6 | 0.00 |
| B01 | 0.816 | 100.3 | 2277 | 595.5 | 0.00 |
| FP101 | 0.06 | 91.05 | 3250 | 938.5 | 3.91 |
| FP102 | 0.277 | 148.35 | 2319 | 612.8 | 0.00 |
| FP103 | 0.379 | 149.92 | 1903 | 474.5 | 0.00 |
| JB01 | 0.351 | 6122.38 | 2343 | 597.5 | 0.00 |
| LQ105 | 4.315 | 61.29 | 2395 | 528.3 | 5.89 |
| LQ107 | 0.153 | 7428.23 | 2440 | 566.7 | 0.00 |
| LQ109 | 0.263 | 1883.03 | 956 | 473.6 | 0.00 |
| U01 | 2.639 | 26.45 | 2719 | 720.4 | 8.25 |
| 655 | 0.238 | 43.56 | 2496 | 612.8 | 1.36 |
| 656 | 0.063 | 34.82 | 2169 | 381.3 | 0.00 |
| 742 | 0.075 | 96.59 | 2070 | 359.2 | 0.81 |
| 743 | 0.033 | 531.19 | 2845 | 771.3 | 0.00 |
| 744 | 0.067 | 4828.76 | 2093 | 504.3 | 0.00 |
| SY07 | 3.118 | 48.24 | 2018 | 366.0 | 0.99 |
| LR01 | 0.11 | 40.92 | 1586 | 397.7 | 1.92 |
